# Squamous Papilloma on Hard Palate: Case Report and Literature Review

**DOI:** 10.5005/jp-journals-10005-1519

**Published:** 2018-06-01

**Authors:** Penmatsa Chaitanya, Satyam Martha, Ramachandran Punithvathy, Madhusudhan Reddy

**Affiliations:** 1 Reader, Department of Pedodontics and Preventive Dentistry, Vishnu Dental College, Bhimavaram, Andhra Pradesh, India; 2 Reader, Department of Pedodontics and Preventive Dentistry, Lenora Institute of Dental Sciences, Rajahmundry, Andhra Pradesh India; 3 Reader, Department of Pedodontics and Preventive Dentistry, Lenora Institute of Dental Sciences, Rajahmundry, Andhra Pradesh India; 4 Reader, Department of Oral Pathology and Microbiology, Army College of Dental Sciences, Secunderabad, Telangana, India

**Keywords:** Human papillomavirus, Koilocytes, Squamous papilloma.

## Abstract

Most of the lesions in the oral cavity have papillary appearance. Oral squamous papilloma (SP) is one such type, which is a benign proliferation of the stratified squamous epithelium and presents as papillary or verrucous exophytic mass induced by human papillomavirus (HPV). Most of the oral mucosal lesions are often asymptomatic and have small progression. The common sites of occurrence include tongue, soft palate, and uvula. Squamous papilloma arising on hard palate is described in this article. Surgical excision of the lesion was done and sent for histopathological analyses that confirmed the clinical diagnosis. In larynx and trachea, malignant transformation of papillomas has been reported. The potentially malignant nature of SP if present needs to be explored.

**How to cite this article:** Chaitanya P, Martha S, Punithvathy R, Reddy M. Squamous Papilloma on Hard Palate: Case Report and Literature Review. Int J Clin Pediatr Dent 2018;11(3):244-246.

## INTRODUCTION

Oral SPs appear as verrucous growth. They present as exophytic mass in the oral cavity induced by HPV. They are benign proliferations of the stratified squamous epithelium.

The World Health Organization defines papilloma as “a range of localized hyperplastic exophytic and polypoid lesions of hyperplastic epithelium with a verrucous or cauliflower-like morphology.”^1^ The sites of predilection include tongue, soft palate, and uvula, but any surfaces of the oral cavity can be affected.^2,3^ Squamous papillo-mas are reported frequently in children but it may affect any age group; 7 to 8% of all oral masses or growths in children comprise oral SP.

It is the fourth most common oral mucosal lesion and is found in 4 of every 1,000 oral soft tissue lesions.^4^ Oral squamous lesions have clinical appearance that mimic exophytic carcinoma, verrucous carcinoma, or condy-loma accuminatum. However, SPs usually have a slow progression and are often asymptomatic and show benign character in pathological examinations.^3^

This article describes a case of SP arising in an unusual location in the oral cavity.

## CASE REPORT

A 10-year-old girl reported with the chief complaint of growth of soft tissue mass on the palate that was present since 1 month. History regarding the growth revealed that it first started as painless slow growing soft tissue mass that gradually enlarged and associated with discomfort and occasional pain on eating and interference while biting. Past medical, dental, and personal history were noncontributory.

**Fig. 1:**
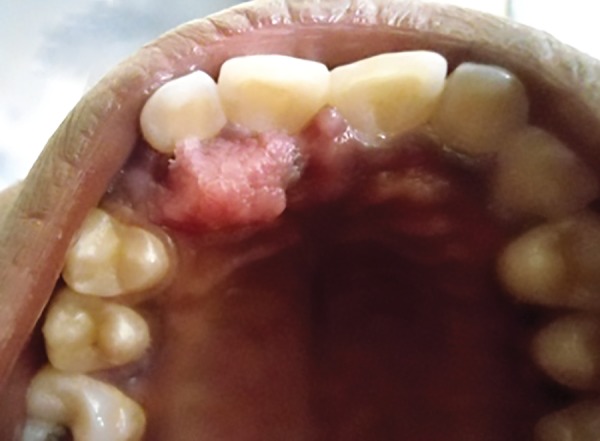
The clinical photograph of the lesion on anterior part of hard palate

**Fig. 2:**
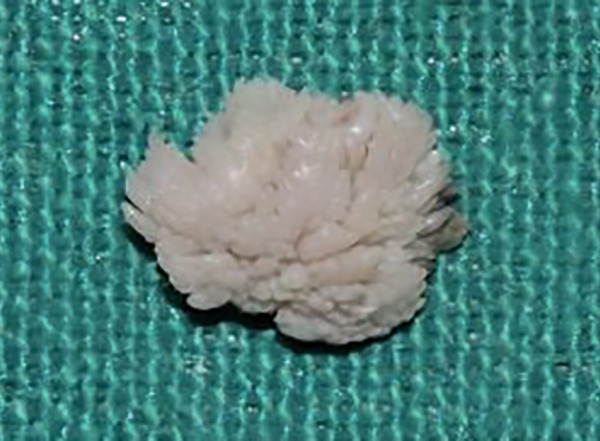
Macroscopic view of excised lesion

General and extraoral examination of the patient did not reveal any significant findings. Intraoral examination revealed a lesion on anteriormost part of hard palate. The lesion was a pale, pink colored, pedunculated growth with finger-like projections of soft tissue present on right side of the rugae area of hard palate just adjacent to incisive papilla. It was approximately 1.5 cm in size, circumscribed, and not associated with bleeding (Figs 1 and 2).

Based on these clinical features, the growth was provisionally diagnosed as papilloma. Surgical excision of the growth was done with a 1 mm margin to the depth of submucosa under local anesthesia. After excisional biopsy, specimen was fixed and stained with hematoxylin and eosin for histological analysis.

**Fig. 3:**
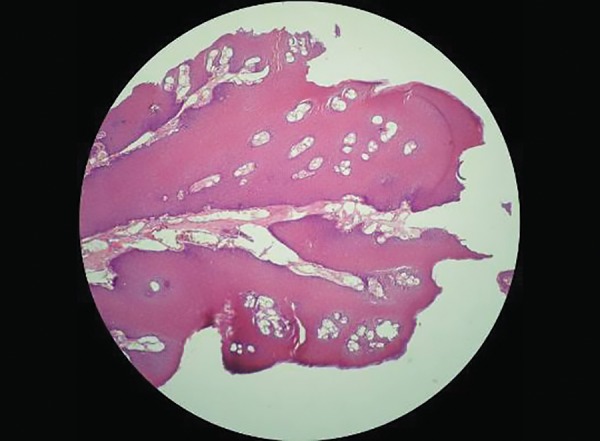
Histopathological view showing proliferation of spinous cells in the form of finger-like projections containing a thin connective tissue core

**Fig. 4:**
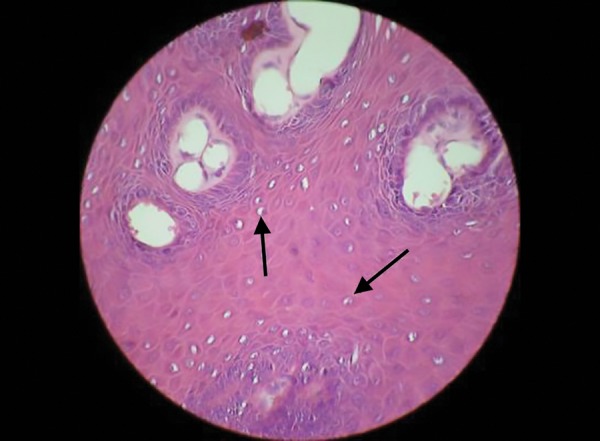
Histopathological view showing hyperplasia of the epithelium. Arrows show the HPV-infected cell (koilocytes)

Histological examination revealed papillary projections of parakeratinized stratified squamous epithelium of variable thickness with localized areas showing mild basilar hyperplasia with few koilocytes and enclosing connective tissue cores (Figs 3 and 4). The connective tissue is fibrocellular in nature with moderate vascularity. Histological features are suggestive of SP.

## DISCUSSION

Oral SPs are benign exophytic lesions presenting as papillary or verruciform proliferation. This lesion was first described by Tomes as gingival “wart” in 1848.^5^ The lesions are softened/flaccid in 66.7% of cases and pedunculated in 75% of the lesions.^2^ Although etiology of papilloma is unknown, it is most commonly associated with HPV 6, 11, and 16.^6^ The HPV is a member of the papovavirus group. It is a deoxyribonucleic acid (DNA) virus containing a single molecule of double-stranded DNA. The virus is a nonenveloped icosahedral particle with diameter ranging from 45 to 55 nm and has 72 cap-someres in a skewed arrangement. Various species are distinct antigenetically sharing some common antigenic determinant. Replication of HPV occurs within the nuclei of epithelial cells in the spinous layer due to stimulation of host DNA synthesis. This induces a series of proliferative alterations that result in tumor growth. Controversy remains on viral transmission pathways in children, although the possibilities include sexual abuse, perinatal transmission, autoinoculation, heteroinoculation, and possibly indirect transmission via fomites.^7^

The location of the lesion and clinical presentation in the present case are similar to common identifications of oral SP. The histopathological examination of the lesion confirmed the clinical diagnosis. The lesions are asymp-tomatic^8^ most commonly as seen in the present case.

Squamous papillomas are traditionally divided into two types: Isolated-solitary and multiple-recurring. Although the isolated-solitary type of SP is common in adults, the clinical presentation of SP was similar to isolated-solitary type in this child. The differential diagnosis of solitary type of oral SP includes verruciform xanthoma, papillary hyperplasia, and condyloma acuminatum.

The clinical appearance of verruciform xanthoma is similar to squamous papilloma, but the lesion has predilection for the alveolar ridge and gingiva. For inflammatory papillary hyperplasia, a cause-and-effect relationship (e.g., lesion appearing under ill-fitting denture) should be evident. On comparison with papilloma, condyloma are larger, have a broader base, and would appear pink-to-red due to less keratinization. Clustered or multiple SPs suggest focal epithelial hyperplasia (Heck disease).^8^

The microscopic appearance of papilloma shows proliferation of the spinous cells in the form of long thin finger-like projections extending above the surface of the mucosa containing a thin connective tissue core. The connective tissue is continuous with stroma of the stalk, the body of the mass, and the surface projections. The epithelial surface may reveal hyperparakeratosis. Basilar hyperplasia and mild mitotic activity may be present occasionally. This should be considered with caution as it can mislead to a diagnosis of mild epithelial dysplasia. There have also been reports of superimposed mycotic infec-tions.^2^ The most characteristic feature of HPV infections is the presence of koilocytes. Koilocyte is an HPV-infected squamous epithelial cell. The cellular changes/cytopathic effects observed are enlargement of the nucleus (two to three times normal size), irregularity of the contour of nuclear membrane, darker than normal staining pattern in the nucleus, and the presence of perinuclear halo.

In a study conducted by Carneiro et al,^2^ koilocytes were demonstrated in 100% of the oral SP studied. Even though SP is a HPV-induced lesion, its infectivity is much less pronounced. Malignant transformation of papillomas in areas, such as larynx and trachea has been reported.^1^

Treatment of choice for SP is surgical excision. Vaccines could be used as adjuvant treatment following surgery by generating an immune response. A decreased prevalence of SP may result in future, against types 6 and 11. Two vaccines have been developed: Cervarix and Gardasil that can prevent infections and precancerous lesion caused by HPV infection.^9^

## CONCLUSION

Though SPs are common lesions of the oral cavity, their occurrence on the hard palate is rare, and its histopatho-logical features overlap with a number of other verru-copapillary lesions. Hence, differentiating papillomas from other lesions is important. With papillomas in other regions showing malignant transformations, the potentially malignant nature of SP if present should be explored.
